# Intravenous Infusions of Glycerol Versus Propylene Glycol for the Regulation of Negative Energy Balance in Sheep: A Randomized Trial

**DOI:** 10.3390/ani9100731

**Published:** 2019-09-26

**Authors:** Mugagga Kalyesubula, Alexander Rosov, Tamir Alon, Uzi Moallem, Hay Dvir

**Affiliations:** 1 Institute of Animal Science, Volcani Center—ARO, 68 Hamakkabim Rd., Rishon LeZion 7528809, Israel; 2 Department of Animal Science, the Hebrew University of Jerusalem, Rehovot 7610001, Israel

**Keywords:** pregnancy toxemia, ketosis, hyperketonemia, hypoglycemia, glycerol, propylene glycol

## Abstract

**Simple Summary:**

Propylene glycol (PG) and glycerol are common energy substances used to supplement the feed of transitioning ruminants in order to minimize the development of metabolic disorders related to energy deficiency. Their effects on the energetic status of the animal have been, thus far, studied mostly by oral administration, which exposes them to substantial microbial metabolism in the rumen. This study compared the direct metabolic effects of these substances following their intravenous (IV) infusion. We found that glycerol was highly glucogenic and insulinotropic, as expected. However, surprisingly, PG had no significant effect on the circulating levels of glucose or insulin. Unlike glycerol, PG significantly raised circulating lactate levels and showed some potential tissue damage activity. Our study points to glycerol, rather than PG, as a potential IV treatment for efficient relief of hypoglycemia and hyperketonemia.

**Abstract:**

Negative energy balance (NEB) is a state of insufficient dietary-energy consumption, characterized by the breakdown of adipose fat to meet the physiological energy expenditure. Extensive NEB, as common in high-yielding transitioning ruminants, drives significant metabolic disturbance and pathologies such as pregnancy toxemia and ketosis. Strategies to minimize the severity of NEB include the use of energy-dense feed supplements, like glycerol and propylene glycol (PG), or IV glucose infusion during severe hypoglycemia. PG and glycerol have been studied mainly by oral or ruminal administration, which exposes them to substantial metabolism in the digestive system. To investigate their direct benefits to mitigating NEB, we intravenously infused them into sheep induced into NEB by feed restriction. Sixteen 5-month-old ewe lambs at NEB were IV-treated with 170 mL isotonic saline containing 15% glycerol or 15% PG. Both PG and glycerol effectively reduced hyperketonemia by 57% and 61%, and inhibited adipose lipolysis by 73.6% and 73.3%, respectively. Surprisingly, only glycerol was glucogenic (*p* < 0.0001) and insulinotropic (*p* < 0.0075), while PG was primarily utilized for production of lactate (*p* < 0.0001). Tissue-damage biomarkers indicated hemolytic activity for PG. This study revealed glycerol as a superior IV treatment for effective relief of NEB. Since it carries no risk of glucose overloading, glycerol IV infusion may also have clinical advantages over glucose for treatment of pregnancy toxemia and ketosis.

## 1. Introduction

The physiological response to insufficient dietary-energy consumption compared to energy expenditure involves adipose lipolysis to meet with the physiological energy demands. Most of the fat catabolism takes place in the liver, and its mobilization from the adipose tissue is mediated by circulating non-esterified fatty acids (NEFA). Such a physiology of negative energy balance (NEB) develops during starvation or fasting [1], and it is very common in ruminants during the transition period from late gestation to early lactation, when their energy demands peak and often exceed those consumed in the diet [2]. However, extensive NEB accompanies hypoglycemia hyperketonemia and can drive major energy-deficiency metabolic disorders such as pregnancy toxemia and lactation ketosis [3].

In the liver, NEFA can be fully degraded, initially via β-oxidation into acetyl-CoA, which can be further metabolized to complete oxidation in the tricarboxylic acid (TCA) cycle to yield the maximum metabolizable energy. However, during states of hypoglycemia and glucose shortage, the oxidative capacity of the TCA cycle is limited. Therefore, partial NEFA oxidation dominates and more of the resulting acetyl-CoA is diverted to hepatic ketogenesis and lipogenesis [4]. Glucose insufficiency and excessive adipose lipolysis may, therefore, lead to hyperketonemia and fatty liver, which are considered the underlying causes of pregnancy toxemia and lactation ketosis [3]. Unlike lactation ketosis, pregnancy toxemia that is prevalent in prolific breeds of sheep and goats [5,6] is rarely reversible and can be fatal [7]. Accordingly, enrichment of the metabolizable energy proportion of the ration during the transition period is a critical element in intensive farming, with the clinical goals of minimizing hypoglycemia and hyperketonemia. Since the dry matter intake of ruminants decreases in pregnancy [8], the use of energy-dense supplements rich in glucogens, often as liquids and/or in semi-solid molasses forms [9,10], has become integral to ruminant nutrition under intensive and semi-intensive management systems.

Glycerol and propylene glycol (PG) are commonly used, as cost-effective glucogens, to supplement the diets of ruminants [11,12,13]. Both reduce NEB associated with the transition period and reduce the risk for pregnancy toxemia and ketosis [12,14,15]. Metabolic effects of glycerol and PG have been mostly studied following administration via drenching, as a feed-top dress or via ruminal infusion of cannulated animals [13,15,16]. In vitro studies revealed that glycerol is metabolized by rumen microbes mainly to propionate, butyrate, and acetate [12,17,18], whereas PG is mainly metabolized into propionate, propanol, and propanal [19,20].

Despite the intensive capacity of the rumen to metabolize glucogens, substantial proportions of large doses of glucogens escape microbial fermentation and are absorbed intact [11,21]. Therefore, the physiological effects of oral treatments with glucogens (e.g., glycerol and PG) result from both their absorption as intact molecules, and as products of their metabolism by microbes in the rumen.

Administration of glycerol and PG into the abomasum of dairy cows revealed contrasting results, with glycerol being superior in increasing concentrations of glucose in blood [22]. Yet studies of the direct contributions of these glucogens, unaffected by the digestive system, have not been reported for small ruminants. To compare the direct effects of glycerol and PG on NEB in sheep, with minimal interference from the digestive system, we employed intravenous (IV) administration of equicaloric doses of glycerol and PG in sheep homogeneously induced into NEB by feed restriction.

This approach yielded outstanding sensitivity to distinguish between the metabolic impacts of glycerol and PG on NEB, which may have implications for the development of new therapeutic protocols to mitigate energy-deficient metabolic disorders of transitioning ruminants.

## 2. Materials and Methods

### 2.1. Animal Studies and Experimental Design

The Volcani Animal Care Committee approved all procedures involving animals in this study (Dossier # 717/17IL). The experiments were conducted with sheep at the Volcani Experimental Farm, Rishon LeZion, Israel, in October 2017. Instead of working with pregnant ewes that vary in many parameters relevant to their energetic status, we chose to work with a homogeneous population of non-pregnant ewe lambs uniformly induced into NEB by feed restriction. There were eight animals per each of the two treatments (*N* = 16 total ewe lambs). This study was designed based on results of a pilot experiment with six ewe lambs per treatment (Figure S1) which showed that the average maximal glucose response to glycerol was more than 2.5 times that for PG (27.3 vs. 10.7 mg/dL, SEM 3.04, *p* < 0.0015). Using the standard deviations obtained from that experiment (10.5 mg/dL) in a retrospective sample-size design calculation in JMP (employing two-sample means power calculation), with a desire to detect a clinical difference of 16.7 mg/dL, a significance level of 0.05 and a power of 80% suggested a total number of 15 animals. Together with the high statistical significance obtained in the pilot experiment with only 12 animals, the use of 16 animals was predicted to safely meet the goals of the current comprehensive study and its detailed analyses. 

To generate comparable intervention groups, with minimized animal selection and confounding factors, a careful randomization procedure was carried out. Ewe lambs (*N* = 16) of the Afec-Assaf breed [23] at 4.5 ± 0.3 SD (standard deviation) months old and 44.3 ± 3.4 SD kg in body weight were assigned to two treatment groups (*n* = 8). To maintain similar body weights of ewes between treatments, a stratification approach was taken by first randomly selecting 8 ewe lambs from our population of 83 ewe lambs born in the same period of the year using the RAND command in Excel. An additional 8 ewe lambs were selected from that pool to most closely match with the weights of the first randomly selected lambs, thereby yielding 8 lamb pairs of similar weights. Then, ewe lambs from each pair were randomly assigned to one of the two treatments. In total, 16 animals were used in the experiment and none were deleted from the study. All ewes were housed in the same pen during the experiment. Animals treated with glycerol were labeled as G7–G12, G15, and G16, and those with PG as P1–P6, P13, and P14. The experiment was not blinded since none of the measurements were subjective.

Typically, lambs at the Volcani experimental flock are weaned at 1.5 months of age and raised under intensive management (fed a ration containing 8% wheat hay, and 92% grain concentrate at 16% protein content) to maximize growth and weight gain (averaged at 300 gr/day) [24]. To induce NEB, the animals were fed only wheat straw *ad libitum* for 2 days, followed by another day of fasting and free access to drinking water. On the morning of the fourth day, when ewes had subclinical hyperketonemia and hypoglycemia levels, IV treatments were initiated with infusions of either glycerol or PG. Based on the results from the pilot experiments, it was determined that concentrations of glucose and β-hydroxybutyrate (BHBA) return to baseline values within about 5.5 h after treatment; therefore, blood sampling in this study occurred over 6.3 h post-treatment. At the end of the experiment, the animals were returned to their normal diet gradually over a period of 3 days.

A single indwelling catheter (Delta Med s.p.a., Viadana, Italy) was installed into the jugular veins of each ewe the day before the experiment began. To prevent coagulation of blood in the catheter, 3–6 mL of 10 IU/mL heparin (LEO Pharma A/S, Ballerup, Denmark) in sterile 0.9% saline (Teva Medical Ltd., Ashdod, Israel) was used to flush the catheter. Each sheep was infused with 170 mL of 0.22 mm filter-sterilized 15% glycerol or 15% PG solutions in isotonic saline. These doses were identified in our pilot experiments to significantly increase concentrations of glucose in the blood. PG was kindly provided as a gift from Biovac (100% PG, Akiva, Israel). Glycerol was purchased (Bio-Lab Ltd., Jerusalem, Israel). 

Blood samples of 5 mL were collected from each animal into heparinized vacutainers (Becton Dickinson and Co., Franklin Lakes, NJ, USA) immediately before treatments and at 15, 40, 60, 140, 200, 270, 330, and 380 min after initiation of treatments. Blood concentrations of glucose and BHBA were determined using the FreeStyle Optium glucometer (Abbot Diabetes Care Ltd., Oxfordshire, UK) [25,26]. To harvest plasma, the heparinized blood was centrifuged at 2000× *g* for 15 min at 4 °C. The supernatants (plasma) were immediately stored at −20 °C until further analyses. 

### 2.2. Plasma Biochemical Analysis

Determinations of lactate, lactate dehydrogenase (LDH), aspartate aminotransferase (AST), alanine aminotransferase (ALT), and bilirubin in plasma were performed using the Cobas C 111 analyzer (Roche Diagnostics, Rotkruez, Switzerland). Plasma insulin was measured using a commercially available ELISA kit (Mercodia, Uppsala, Sweden), and concentrations of NEFA in plasma were determined using a NEFA kit (Wako Chemicals, GmbH, Neuss, Germany), following the manufacturer’s instructions.

### 2.3. Statistical Analysis

Data of continuous dependent variables (glucose, BHBA, lactate, NEFA, Insulin, LDH, AST, ALT, and total and conjugated bilirubin) were analyzed via the repeated measures ANOVA with the mixed model and autoregressive approach in JMP (VERSION 14.0.0, SAS Institute Inc., Cary, NC, USA). The model included three fixed factors (Treatment, Time, and Treatment × Time), one random factor (Animal) nested within the Treatment variable, and two covariates (pretreatment values and body weights). The Treatment had two levels (glycerol and PG), and the Sampling Time from onset of treatment was treated as a nominal variable of 8 levels. The distribution of the model residuals was visually validated for normality. Comparisons between the treatments at specific sampling times (as indicated in the figure legends) were assessed using contrast *t*-tests and the reported *p*-values were Bonferroni-corrected to account for the multiple comparisons performed.

The area under the curve (AUC) was determined using the trapezoidal rule, computed as the total area between the response curve and the baseline. The baseline for each curve was obtained from a linear line connecting the first and the last measured values. Differences between AUC and/or delta values (the difference between the peak and the pretreatment values in percentage) were analyzed by the standard least squares fit model in JMP, with pretreatment values and body weights as covariates. Significance was accepted at *p* < 0.05. The effect of body weights of ewes on their AUCs was not significant (*p* < 0.419, as tested via standard least squares); therefore, they were not included in the final statistical model.

## 3. Results

As a first step in studying the effects of IV treatments with glycerol and PG on the energetic status of sheep, all 16 ewe lambs were induced into NEB. This resulted in moderate but steady hyperketonemia, with an average concentration of BHBA in the blood of 0.71 ± 0.17 mM, and a state of relative hypoglycemia with average blood glucose values down to 68% of their non-fasting basal values (56.7 ± 5.9 vs. 82.7 ± 6.1 mg/dL, respectively). 

All of the measured energetic-state-related factors varied with the Sampling Time (*p* < 0.006; Table 1), as expected from the administration of a single bolus of energy. Therefore, the responses of these factors were evaluated based on the AUC of their concentrations as a function of Sampling Time, to obtain more inclusive and statistically valid analyses of the effects of treatments [27]. The maximal value of the response minus the pretreatment value (referred to as delta and expressed as a percentage of the pretreatment values) was another informative measure of the effects of the treatments. 

### 3.1. Glycerol and Propylene Glycol Were Both Anti-Ketogenic, but Only Glycerol was Glucogenic 

**Figure 1:**
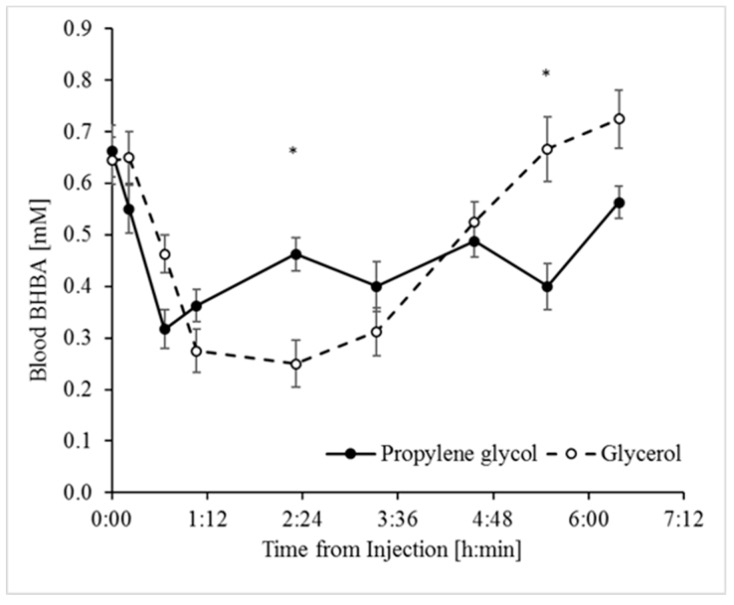
BHBA response to the propylene glycol and glycerol treatments. Analyses indicate significant effects of Treatment, Time, and Treatment × Time interaction (Table 1). * Significant difference at α = 0.00625 after Bonferroni correction. SEM = 0.03. Values represent mean ± SE.

Following the IV treatments, concentrations of BHBA in blood decreased by 61% (with glycerol) and 51% (with PG) of their initial subclinical levels (Figure 1). Notably, the response to PG was faster, reaching a minimum within 40 min of treatment compared to a minimum at 140 min following the glycerol treatment. However, the overall response had a more pronounced bell-shaped curve for glycerol (Figure 1). Despite the kinetic differences between the BHBA responses, the overall magnitude of the effect, which is better represented by the AUC, was not significantly different between the treatments (Table 2), indicating that both PG and glycerol were efficiently utilized to reduce ketogenesis.

**Figure 2:**
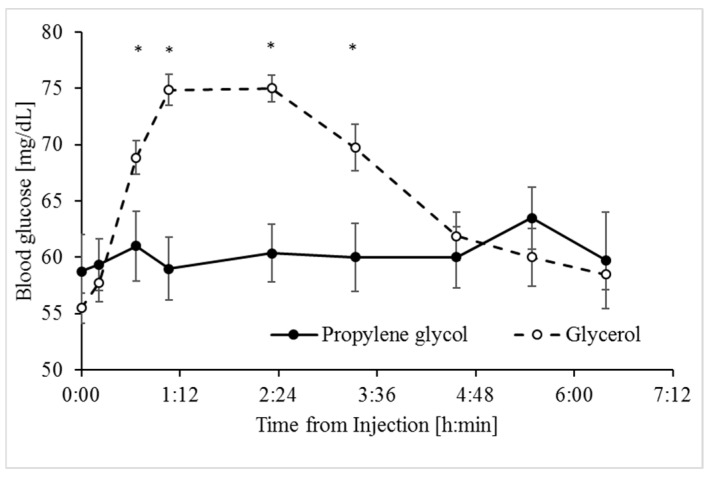
Glucose response to the propylene glycol and glycerol treatments. Analyses indicate the effects of Treatment, Time, and Treatment × Time interaction (Table 1). * Significant difference at α = 0.008 after Bonferroni correction. SEM = 0.974. Values represent mean ± SE.

Unlike its similar effects on hyperketonemia, the glycerol treatment induced a significantly greater glucose response than the PG treatment (Figure 2), as indicated by their respective AUC and delta values (Table 2). Blood glucose levels increased immediately following the glycerol treatment and peaked within 1 h, while in response to the PG treatment, concentrations of glucose in blood remained stable around basal values (delta of 42.3% and 11.2%, respectively; *p* < 0.0001, Table 2).

### 3.2. Unlike Glycerol, Propylene Glycol Increased Circulating Lactate Concentrations 

**Figure 3:**
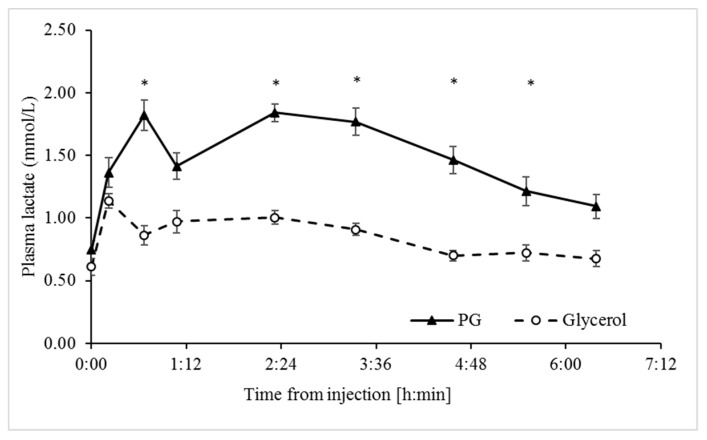
Lactate response to the propylene glycol (PG) and glycerol treatments. Analyses indicate the effects of Treatment, Time, and Treatment × Time interaction (Table 1). * Significant difference at α = 0.00625 after Bonferroni correction. SEM = 13.2. Values represent mean ± SE.

The plasma lactate response was lower in response to the glycerol treatment than the PG treatment, based on significant differences in both the AUC and delta values (*p* < 0.0001; Table 2). Immediately following the PG treatment, the plasma concentrations of lactate increased sharply and peaked within 40 min, whereas with the glycerol treatment, concentrations of lactate remained relatively low throughout the sampling period (Figure 3).

### 3.3. Glycerol Stimulates Greater Release of Insulin into Plasma than Propylene Glycol

**Figure 4:**
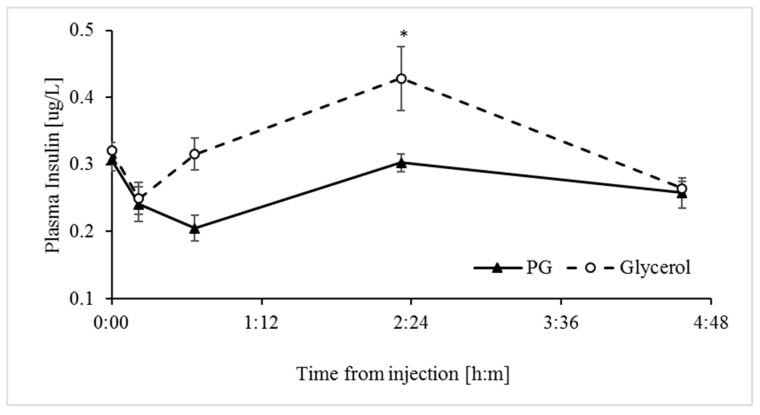
Insulin response to the propylene glycol and glycerol treatments. Analyses indicate the effects of Treatment and Time (Table 1). * Significant difference at α = 0.0125 after Bonferroni correction. SEM = 0.01. Values represent mean ± SE.

Among the multitude of anabolic actions induced by insulin to balance nutrient availability and demands, insulin also down-regulates adipose lipolysis and hepatic ketogenesis. Therefore, to better understand the effects of the glucogenic treatments on hypoglycemia and hyperketonemia, we determined simultaneous effects on circulating concentrations of insulin. The glycerol treatment induced a greater plasma insulin response (*p* < 0.0075) than the PG treatment based on the AUC values (Table 2). Concentrations of insulin peaked 2 h following the glycerol treatment, but they did not increase (*p* > 0.1) above baseline following the PG treatment (Figure 4).

### 3.4. Both Glycerol and Propylene Glycol are Anti-Lipolytic, but the Response to Glycerol is Faster 

Energy stored in adipose tissue is delivered to tissues primarily in the form of NEFA [4,28,29]. Therefore, increased concentrations of NEFA in the plasma indicate adipose lipolysis, which is a key feature of NEB.

**Figure 5:**
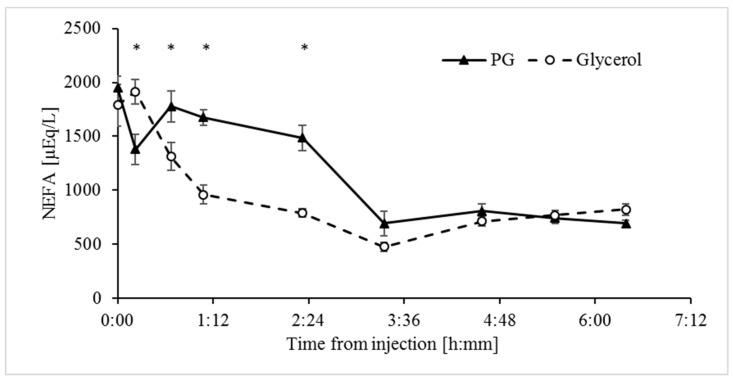
Non-esterified fatty acid (NEFA) response to the propylene glycol and glycerol treatments. Analyses indicate the effects of Treatment, Time, and Treatment × Time interaction (Table 1). * Significant difference at a = 0.00625 after Bonferroni correction. SEM = 53. Values represent mean ± SE.

Both glycerol and PG decreased (*p* < 0.0001) concentrations of NEFA in plasma within 3.5 h to 73% and 71% of the baseline values, respectively (Table 2). However, the NEFA response to the glycerol treatment was much faster (Figure 5), with a substantial decrease within 60 min of treatment compared to a substantial decrease at 200 min after the PG treatment. Overall, the effect of the glycerol treatment on the concentrations of NEFA in plasma was greater up to 140 min after treatments, but NEFA values were then similarly low for both treatment groups (Figure 5). 

### 3.5. Biomarkers of Liver Function Indicate that Unlike Glycerol, Propylene Glycol may Induce Tissue Damage and Hemolysis

**Figure 6:**
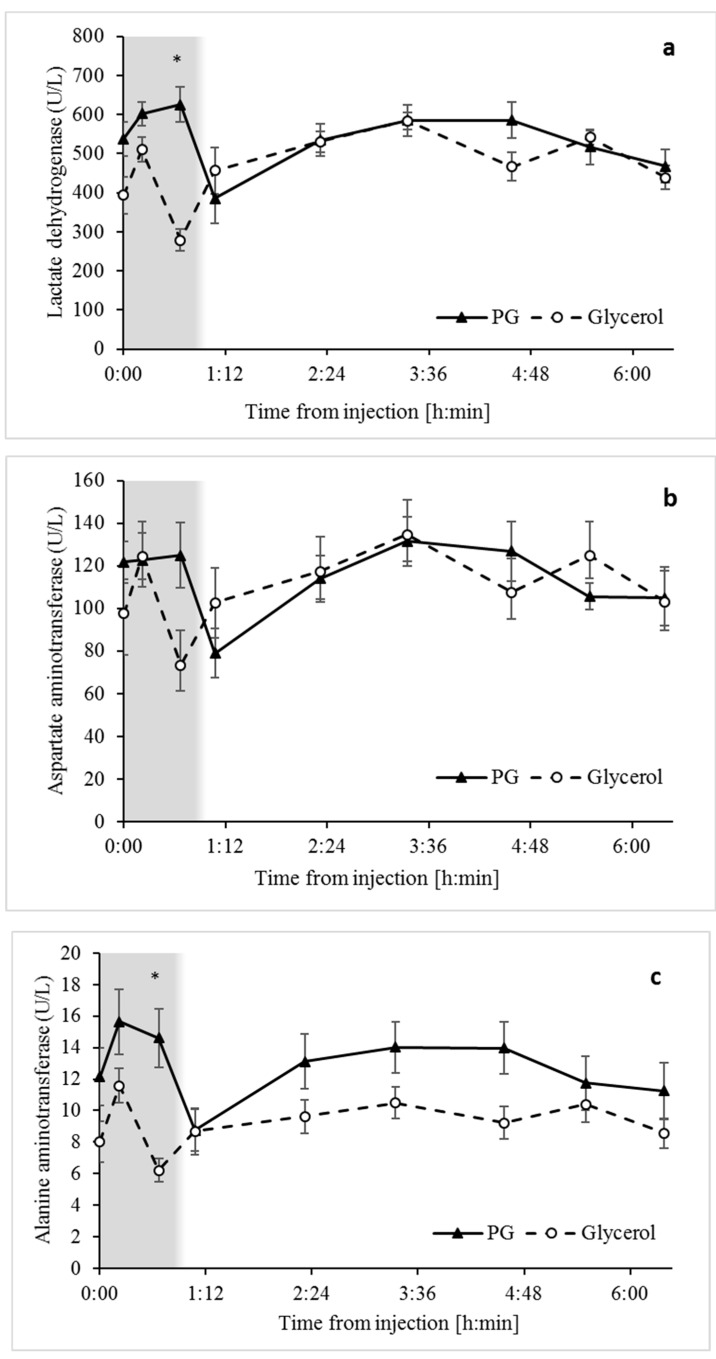
Liver and tissue damage biomarkers in response to intravenous treatments with PG and glycerol. (**a**) Lactate dehydrogenase (LDH) increased to a greater extent in response to PG compared to glycerol (*p* < 0.0001). This difference extended only to the first 40 min after treatment (shaded) and not to the entire sampling time. Analyses reveal the effects of Time and Treatment × Time interaction (Table 1). SEM = 25.3. A similar pattern of change was observed for (**b**) aspartate aminotransferase (AST), which also increased in response to PG for the first 40 min after treatment (shaded) (*p* < 0.01), with a fixed effect of Time and Treatment × Time interaction (Table 1). SEM = 10.7. (**c**) Alanine aminotransferase (ALT) also increased in response to PG during the first 40 min after treatment (*p* < 0.0005), with a fixed effect of Time and Treatment × Time interaction. SEM = 1.3. Graphs of (**a**), (**b**), and (**c**) represent mean ± SE values. *p*-values are considered significant if lower than the Bonferroni-corrected significance (a = 0.00625). * Significant difference.

**Figure 7:**
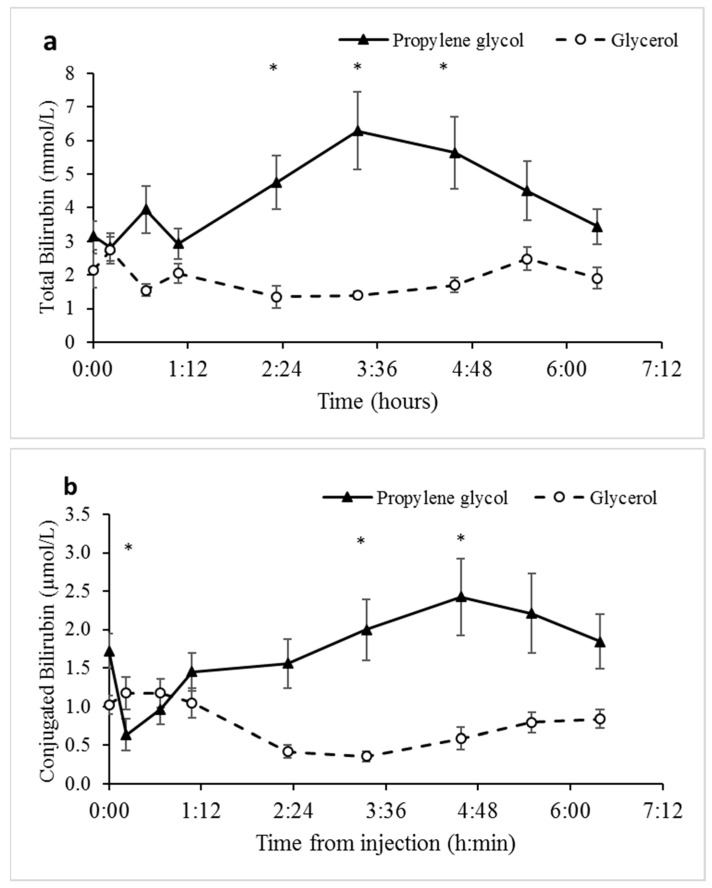
Concentrations of bilirubin as a function of Time from the propylene glycol and glycerol treatments. (**a**) Total bilirubin. Analysis indicates effects of Treatment, Time, and Treatment × Time interaction (Table 1). SEM = 0.192. Values represent mean ± SE. (**b**) Conjugated bilirubin. Analysis indicates effects of Time and Treatment × Time interaction (Table 1). SEM = 0.192. Values represent mean ± SE. * Significant difference at α = 0.00625 after Bonferroni correction. The difference in values for total (**a**) and conjugated (**b**) bilirubin are substantial. Specifically, unconjugated bilirubin accounts for a significant portion of the total bilirubin, which is highly indicative of increased hemoglobin catabolism and intravascular hemolytic activity [32].

The enzymes LDH, ALT, and AST from liver and other tissues, such as heart and muscle [30,31], increase in plasma following tissue damage and are commonly used as biomarkers of liver and tissue damage. Overall, the concentration of LDH, ALT, and AST tended to be higher (Figure 6) in response to the PG treatment, being particularly significant at the 40 min sampling time, after which concentrations of the biomarkers decreased. Since the number of sampling time points within 1 h post-treatment were too few, neither AUC nor delta comparisons were informative. Surprisingly, plasma samples from sheep treated with PG had a significant reddish coloration compared to those from glycerol treated sheep, which had a normal yellowish color (Figure S2). Consistent with this evidence for hemolytic activity, concentrations of bilirubin in plasma were also greater (*p* < 0.007) for PG- versus glycerol-treated sheep (Figure 7 and Table 1).

## 4. Discussion

For several decades, glycerol and PG have been used widely as oral glucogenic supplements to address metabolic disorders, such as pregnancy toxemia and lactation ketosis—both of which develop in the background of NEB [33,34]. To the best of our knowledge, this is the first report of a study to compare the effects of their intravenous administration on the physiology of NEB in domestic ruminants.

Despite the natural development of NEB in transitioning prolific ewes, they present with varying degrees of hypoglycemia and hyperketonemia, as well as of age, weight, litter size, parity, and history of morbidities. The resulting substantial variability among pregnant ewes with respect to many of the key factors related to their energetic state limits the sensitivity of the evaluation of the effects of glucogens on physiological parameters of interest. To minimize this among-animal variability, we used ewe lambs of the same breed, age group, and of similar body weights, uniformly induced into NEB to assess effects of glycerol and PG as therapeutic treatments to mitigate adverse effects of hypoglycemia and hyperketonemia. 

In line with abomasum infusion studies in dairy cows [22], which bypasses fermentation by rumen microbes, the IV glycerol treatment in sheep increased blood glucose to a greater extent than the PG treatment (Figure 2). The glycerol treatment may have reduced the hyperketonemia via several mechanisms. First, the substantial increase in glucose availability increases the abundance of 4-carbon intermediates of the TCA cycle, affecting more directly oxaloacetate levels [35]. Consequently, the capacity of oxidation of acetyl-CoA via the TCA cycle increases and this depletes precursors for the synthesis of ketone bodies [11,36]. Second, the significant stimulation of release of insulin into plasma by glycerol should signal directly for inhibition of ketogenesis [37] through mTORC1 inhibition of peroxisome proliferator-activated receptor gamma (PPARγ) [38]. Based on the kinetics of the insulin and glucose responses to the glycerol treatment (Figure 2 and Figure 4), the insulinotropic properties of glycerol appear secondary to the increase in blood glucose. In line with this and unlike the effects of glycerol, the PG treatment had no significant effect on concentrations of plasma insulin (Figure 4), likely due to its poor conversion to glucose (Figure 2). The insignificant increase in insulin following the PG treatment is in contrast to previous results from studies in which PG was introduced into the rumen of dairy cows [20,22]. However, this apparent discrepancy can be attributed to the metabolism of PG to insulinotropic metabolites such as propionate by microbes in the rumen [12,39]. Propionate is the main physiological precursor for glucose in ruminants. Third, inhibition of lipolysis in adipose tissue by the glycerol treatment, most likely due to stimulation of insulin release [40,41] (Figure 4), resulted in a significant decrease in plasma NEFA (Figure 5). Since NEFA are used by the liver as a principal fuel for hepatic ketogenesis [29,42,43], the efficient reduction in plasma NEFA would contribute to the observed reduction in hyperketonemia. 

Mammals metabolize PG to lactate via hepatic alcohol and aldehyde dehydrogenases [44]. The conversion of lactate to pyruvate can fuel both hepatic gluconeogenesis and oxidative metabolism. Moreover, like glucose, lactate can directly support the fetal placental unit during pregnancy [2]. In contrast to PG, glycerol can serve as a source for lactate only indirectly. Namely, it enters gluconeogenesis via the glycolytic pathway to yield pyruvate that can then be converted to lactate via lactate dehydrogenase. However, at insufficient concentrations of glucose in blood, as in NEB physiology, this pathway is not expected to be significant since the rate of glycolysis is too low [45]. Consistently, our data show that the lactate response to glycerol was minor compared to the substantial response to PG (Figure 3).

Interestingly, the metabolism of PG to pyruvate through lactate [46] provides for a shorter biochemical path than that for glycerol to increase the abundance of TCA-cycle intermediates, and thus potentially to a faster decline in hyperketonemia. Noticeably, a faster BHBA response in the present study occurred in response to PG as compared to glycerol (Figure 1), and, accordingly, the lactate response to the PG treatment was faster (peaked within 40 min) and more pronounced than for glycerol (Figure 3). Nonetheless, the more robust stimulation of insulin release by glycerol likely contributed substantially to the reduction of hyperketonemia, as revealed by the lower minimum concentrations of BHBA (Figure 1).

The biomarkers for liver health, particularly LDH and ALT, increased during the first 40 min following the PG treatment, but not following the glycerol treatment (Figure 6). Thus, PG may have potential adverse effects via tissue damage. Combined with evidence for hemolysis in plasma following the PG treatment (Figure S2), and the increase in bilirubin (Figure 7), particularly of unconjugated bilirubin that is related to hemoglobin catabolism [32], the potentially toxic activity of PG must be considered when assessing therapeutics for the treatment of hypoglycemia and hyperketonemia. Indeed, hemolytic activity has been reported in sheep due to the use of PG as a drug carrier [47]. The wide use of PG as an energy source and as a carrier for medications calls for further investigations of its safety.

## 5. Conclusions

The results of this study clearly indicate that intravenous treatments with glycerol and PG effectively reduced hyperketonemia and lipolysis. However, no glucogenic or insulinotropic value was found for PG, and its apparent conversion to lactate surprisingly improved only the ketotic, but not the hypoglycemic state. PG treatment also resulted in significant hemolytic activity. Thus, glycerol is a potentially superior intravenous alternative for efficient relief of NEB, with robust benefits to reducing hypoglycemia, hyperketonemia, and adipose lipolysis.

## 6. Patent

A patent application related to the results from this study has been initiated by the corresponding author and its research institute.

## Figures and Tables

**Table 1:** Effects of intravenous infusion of glycerol and propylene glycol (PG).

	Treatment			*p-*Value				
	Glycerol (Mean)	PG (Mean)	SEM	Treatment	Time	Treatment × Time	Animal Effect	Pretreatment
BHBA, mM	0.49	0.46	0.03	0.643	0.0001	0.0001	0.0492	0.0336
Glucose, mg/dL	69.0	59.0	1.14	0.0001	0.0006	0.0005	0.1760	0.0029
Lactate, mmol/L	0.89	1.48	0.075	0.0002	0.0001	0.0001	0.0307	0.8167
Insulin *, µg/L	0.31	0.25	0.01	0.0078	0.0057	0.0884	1	0.4360
NEFA, µEq/L	982	1144	10.7	0.047	0.0001	0.0001	0.09	0.0781
LDH, U/L	498.7	515.7	24.4	0.648	0.0174	0.0013	0.121	0.0178
AST, U/L	117.5	107.1	6.86	0.312	0.0008	0.0018	0.029	0.0003
ALT, U/L	10.9	11.3	0.62	0.710	0.0003	0.0005	0.0401	0.0001
Total Bilirubin, µmol/L	2.25	3.93	0.35	0.0074	0.0123	0.0001	0.05	0.0095
Conjugated Bilirubin, µmol/L	1.05	1.39	0.15	0.174	0.0283	0.0001	0.067	0.0033

Mean: Least squares mean; SEM: Standard error of the mean. * Statistical analysis refers only to the lasting time of the signal (first 4.5 h). Effect of body weight not shown since no significant differences were detected.

**Table 2:** Effect of intravenous infusion of glycerol and propylene glycol (PG) on the percentage delta (Delta %) and area under the curve (AUC) for concentrations of nutrients, metabolites, and hormones in the blood.

	Treatment		SEM	*p-*Value		
	Glycerol	PG		Treatment	Pretreatment	Body Weight
	(Mean)	(Mean)				
BHBA						
Delta %	61	57.6	7.02	0.746	0.215	1
AUC, min × mM	79.1	64.1	15	0.494	0.887	NA
Glucose						
Delta %	42.3	11.2	2.69	0.0001	0.0002	0.0102
AUC, min × mg/dL *	3178.2	239	377.3	0.0001	0.065	NA
Lactate						
Delta %	87.1	192.8	11.1	0.0001	0.0001	0.0227
AUC, min × mmol/L	71.8	239.1	19.2	0.0001	0.236	NA
Insulin						
Delta %	41.7	7.2	10.3	0.0399	0.113	0.735
*AUC, min × µg/L	14. 6	−5.1	3.75	0.0075	0.718	NA
NEFA						
Delta %	73.2	71.4	2.67	0.692	0.0286	0.518
AUC, min × µEq/L	178194.2	62670.9	15064.5	0.0001	0.0001	NA
Total Bilirubin						
Delta %	59.1	155.8	27.2	0.033	0.0109	0.285
AUC, min × µmol/L	−92.78	505.4	147.1	0.0161	0.6839	NA
Conjugated Bilirubin						
Delta %	33.7	61.8	23.8	0.464	0.383	0.582
AUC, min × µmol/L	51.8	25	49	0.735	0.258	NA

Delta % = (peak− pretreatment value) × 100/(pretreatment value). Mean: Least squares mean; SEM: Standard error of the mean; NA: Not applicable * Statistical analysis refers only to the lasting time of the signal (first 4.5 h).

## Supplementary Materials

The following are available online at https://www.mdpi.com/2076-2615/9/10/731/s1, Figure S1: Glucose blood concentration in response to propylene glycol and the glycerol intravenous treatments, Figure S2: Representative plasma samples isolated form sheep treated with glycerol (left) and propylene glycol (right).
