# Supplementary material for: Intravenous Infusions of Glycerol Versus Propylene Glycol for the Regulation of Negative Energy Balance in Sheep: A Randomized Trial
Source: Animals (Basel). 2019 Sep 26;9(10):731. doi: 10.3390/ani9100731 (PMC6826570; doi:10.3390/ani9100731)
Supplement: Supplementary file 1 [file animals-09-00731-s001.pdf]

Supplementary material – Kalyesubula *et al*.

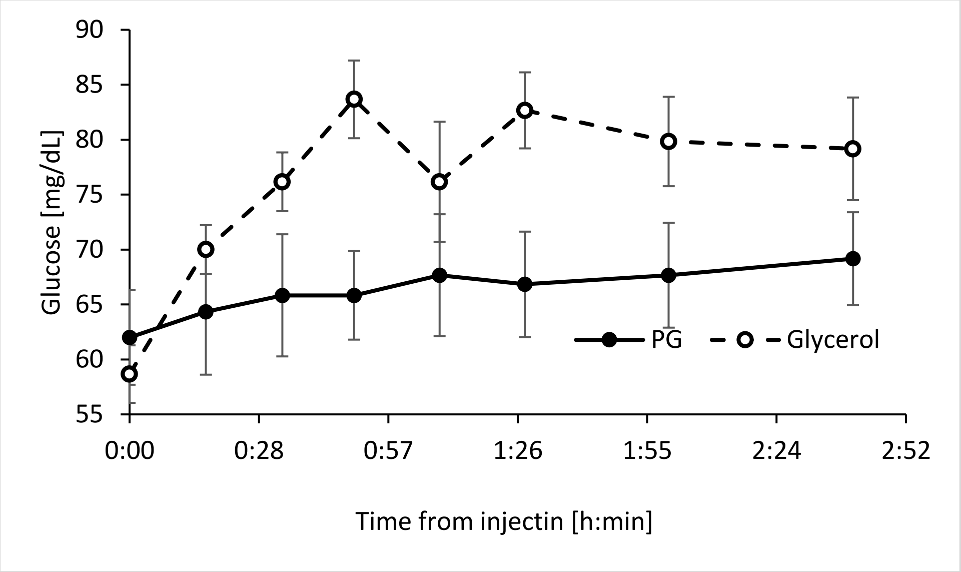

Figure S1. Glucose blood concentration in response to propylene glycol and the glycerol intravenous treatments. Each value represent average (±SE) glucose concentration measured from 6 ewe lambs at eight time points post-injection. 170 ml of 15% PG or glycerol were infused to each animal as described in the M&M. The maximal response, defined by the difference between the pretreatment value to the maximum glucose concentration measured per animal. The average maximal response for glycerol was significantly higher than for PG (27.3 vs. 10.7 mg/dL respectively, $p<0.0015$).

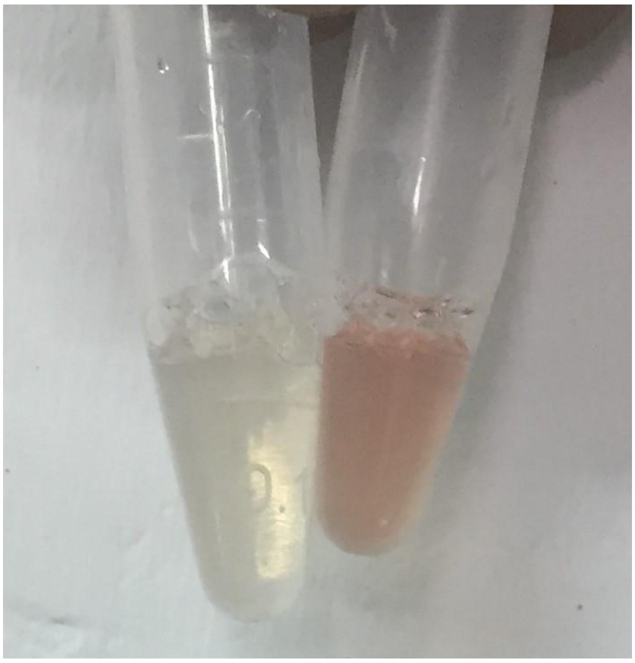

Figure S2. Representative plasma samples isolated form sheep treated with glycerol (left) and propylene glycol (right). The clear reddish coloration of the plasma from the sheep treated with propylene glycol reflects the levels of free circulating hemoglobin. The intense color indicates extensive hemolytic activity.
